# Everybody's Page

**Published:** 1892-10-22

**Authors:** 


### Everybody's Page.

The first Christian building in Tokio was erected 25 years ago. There are now 92 Christian churches and chapels there.

In Finland, above all other countries, do women enter into the business of life. They are clerks, doctors, dentists, builders, managers of small companies, and bank cashiers. They are especially sought for in the last capacity, on account of their reputation for honesty.

A Philadelphia doctor points out the relation between consumption and insanity. Asthma he considers closely allied to phthisis on the one hand, and to insanity on the other. He considers that the link that binds pulmonary phthisis to insanity and to other neuroses is a disease of the vagi.

The French Society of Hygiene has appointed a commission to report on the Cohen system of smoke-consuming chimneys in use in several manufactures in Berlin. They report that the system is efficacious and simple of application. If this is so, may we hope our own sanitary authorities will not be far behind in introducing the method into fog-ridden London?

One by one the maxims of our childhood are reversed by new theories. Who amongst us has not been warned when young by "Do not bathe when heated?" Nowadays we are told "there is absolutely no danger in bathing when heated; on the contrary, this is the best condition to be in when you enter the water." We are told at the same time not to indulge in too long an immersion.

Indian mothers are wiser in one respect than we are. They are most careful to inculcate the habit of keeping their children's mouths shut from their infancy. When a baby is laid down to sleep the mother carefully presses its lips together. The habit thus early acquired seldom departs in after life. Many of us who dwell in London would suffer less from fogs were we to be careful in this particular. Many English mothers regard with little concern an early inclination to keep the mouth open, on the part of their children. It is a great mistake to allow the defect to pass unnoticed, for, if only a matter of carelessness, it may tend to foster a weakness of throat or lungs, and if from some physical cause it should be seen to at once before it is too late to remedy the evil.

Ireland has only eight theatres—three for Dublin, one in Belfast, one in Cork, one in Limerick, one at Waterford, and one for Londonderry.

A Viennese doctor highly recommends hydriate processes in cases of sleeplessness arising from over-pressure of mental work, or in nervous cases. A moist swathing of the body, followed by douche baths, is the main part of the treatment.

If people have ashpits within seven feet of their drinking water cistern, it is small wonder that they do not live to tell the unsavoury tale. Whether the alleged case of Asiatic cholera at March was the disease in its worst form, it is clear that the ground was in the best possible condition to welcome it as a visitor.

The increased interest taken of late years in the question of funeral reform will, it is to be hoped, ultimately bear good fruit. Sentimental obstacles too often bar the path of common sense which might otherwise prevail more speedily. The question is one which cannot be settled by legislation satisfactorily unless backed by a strong public opinion in favour of a general observance of sanitary laws in the disposal of the dead. The suggestion that there should be a Minister of Health is well worthy of consideration on the part of the Government, and it is certain that no advance in burial reform will be made until all authority over burial places is concentrated, say, in the Local Government Board.

Another step towards the industrial emancipation of women, which is by no means outside the bounds of probability, is the admission of them to the ranks of school inspectors. The absurdity of men criticising the needlework, of which the majority must know less than the pupils whose work they are judging, has been pointed out on more than one occasion. There are some things women can do better than men, and this is one of them. It so happens that a school inspector can occasionally fall back on his wife to help him in the difficulty, but every inspector cannot do so. It is sometimes alleged that one of the reasons why women are paid less than men for their labours is that they are insufficiently equipped for life's work, an objection which certainly would not apply in this case.

